# Fatty Acid Desaturases: Uncovering Their Involvement in Grapevine Defence against Downy Mildew

**DOI:** 10.3390/ijms22115473

**Published:** 2021-05-22

**Authors:** Gonçalo Laureano, Ana Rita Cavaco, Ana Rita Matos, Andreia Figueiredo

**Affiliations:** Biosystems & Integrative Sciences Institute (BioISI), Faculdade de Ciências, Universidade de Lisboa, Campo Grande, 1749-016 Lisboa, Portugal; arcavaco@fc.ul.pt (A.R.C.); armatos@fc.ul.pt (A.R.M.)

**Keywords:** *Vitis vinifera*, *Plasmopara viticola*, plant defence, lipid signalling, fatty acid modulation, fatty acid desaturases, biotrophy

## Abstract

Grapevine downy mildew, caused by the biotrophic oomycete *Plasmopara viticola*, is one of the most severe and devastating diseases in viticulture. Unravelling the grapevine defence mechanisms is crucial to develop sustainable disease control measures. Here we provide new insights concerning fatty acid’s (FA) desaturation, a fundamental process in lipid remodelling and signalling. Previously, we have provided evidence that lipid signalling is essential in the establishment of the incompatible interaction between grapevine and *Plasmopara viticola*. In the first hours after pathogen challenge, jasmonic acid (JA) accumulation, activation of its biosynthetic pathway and an accumulation of its precursor, the polyunsaturated α-linolenic acid (C18:3), were observed in the leaves of the tolerant genotype, Regent. This work was aimed at a better comprehension of the desaturation processes occurring after inoculation. We characterised, for the first time in *Vitis vinifera*, the gene family of the FA desaturases and evaluated their involvement in Regent response to *Plasmopara viticola*. Upon pathogen challenge, an up-regulation of the expression of plastidial FA desaturases genes was observed, resulting in a higher content of polyunsaturated fatty acids (PUFAs) of chloroplast lipids. This study highlights FA desaturases as key players in membrane remodelling and signalling in grapevine defence towards biotrophic pathogens.

## 1. Introduction

Grapevine (*Vitis vinifera* L.) is one of the most cultivated fruit plants in the world. *Vitis vinifera* cultivars, used for wine production, are highly susceptible to several diseases, particularly to downy mildew. This disease is caused by the obligatory biotrophic oomycete *Plasmopara viticola* (Berk. et Curt.) Berl. et de Toni, introduced in Europe in the late nineteenth century [1]. To cope with this disease, current strategies rely on the massive use of phytochemicals in each growing season. Understanding the grapevine defence mechanisms is vital to develop alternative and sustainable disease control approaches.

In the last years, lipid composition and associated signalling pathways have been highlighted by their important roles in plant defence mechanisms. These signalling events rely on lipids and lipid-derived molecules, which are major components of cell membranes and an important source of energy. Fatty acids (FAs) participate in plant defence, modulating a number of signal transduction pathways. In response to adversities, changes in membrane lipid composition can alter membrane properties, fluidity and permeability [2]. Moreover, membrane lipids and FAs can act themselves as signalling molecules or can be channelled to biosynthetic pathways of other signalling molecules, such as jasmonic acid (JA) and other oxylipins, derived from polyunsaturated FAs (PUFAs) [3]. In plants, FAs are generated in the chloroplast. This process forms mainly palmitic acid (C16:0) and stearic acid (C18:0) that is immediately converted into oleic acid (C18:1). These FAs are incorporated in plastidial glycerolipids or exported to the endoplasmic reticulum (ER) as carrier protein (ACP) esters for the biosynthesis of extraplastidial lipids [4]. The generation of PUFAs results from the activity of FA desaturases that catalyse the insertion of double bonds into the acyl chains of glycerolipids [5].

Fatty acid desaturases are key enzymes in lipid metabolism since they are responsible for the biosynthesis of unsaturated FAs. These enzymes, which can be soluble or membrane-bound, are classified according to the substrate carrier and the local insertion of the double bond. Regarding their substrate carrier, FA desaturases are subdivided into acyl-ACP, acyl-coenzyme A (CoA) and acyl-lipid desaturases. They can also be distinguished by the terminus of the FA chain, where the double bond is introduced, classified as front-end (Δ) and methyl-end (ω). The Δ desaturases catalyse the formation of a first double bond at the Δ9 position of the acyl group, as well as bonds between pre-existing double bonds and the carboxyl end of the FA chain. The ω desaturases introduce a double bond between the pre-existing double bond and the methyl end of the FA chain [6].

Soluble desaturases are specific enzymes of photosynthetic organisms located in the stroma of chloroplasts. These enzymes introduce a double bond into FA bounded to ACP, being named as acyl-ACP desaturases (SAD, also known as FAB2) [7]. The membrane-bound desaturases are a highly diverse group and can be found in ER [8] or in chloroplast membranes [9]. This group is subdivided into acyl-CoA and acyl-lipid desaturases. The acyl-CoA desaturases, present in animals, yeasts and fungi, introduce double bonds into the FA chain that are bound to CoA. Acyl-lipid desaturases, present in photosynthetic organisms, introduce double bonds into the FA moieties of glycerolipids [10].

The FA desaturase family has already been characterised in some higher plants, such as *Arabidopsis thaliana* [11], *Glycine max* [12], *Gossypium raimondii* [13], *Arachis hypogaea* [14] and *Oryza sativa* [15]. The studies carried out in Arabidopsis mutants, lacking a specific desaturase activity, provided the majority of the available information regarding the function and specificity of each FA desaturase in plants [11]. It was shown that FA desaturation is carried out by SAD and by the membrane-bound acyl-lipid desaturases. SAD (plastidial Δ9) converts C18:0 in C18:1, in the stroma of chloroplasts [16]. The group of membrane acyl-lipid desaturases comprises enzymes with distinct types of cellular location and selectiveness: FAD2 (microsomal Δ12 also called ω6), FAD6 (plastidial Δ12 also called ω6), FAD3 (microsomal Δ15/ω3), FAD7 and FAD8 (plastidial Δ15/ω3), FAD4 (plastidial *trans* Δ3), FAD5 (plastidial Δ7), ADS (microsomal/plastidial Δ9), SLD (sphingolipid Δ8), and DES (sphingolipid Δ4). The desaturation of C16:0 into palmitoleic acid (C16:1) in both the ER and the chloroplast is ensured by the action of different ADS isoforms [17,18]. In *A. thaliana*, the desaturation of C16:0 in C16:1 *trans* is carried out by the action of FAD4 in the chloroplast [19]. FAD2 and FAD6 are responsible for the synthesis of linoleic acid (C18:2) from C18:1 in the ER and chloroplasts, respectively [20,21]. The synthesis of α-linolenic acid (C18:3) from C18:2 is catalysed by FAD3 in the ER [22] and FAD7 and FAD8 in the chloroplast [23]. The introduction of double bonds in sphingolipids is carried out by SLD [24] and DES [25]. SLD leads to the production of 8 (Z/E)-C18-phytosphingenine [24], and DES produces sphinga-4,8-dienine [25].

Our previous works have given rise to evidence that the first hours of interaction between grapevine and *P. viticola* are vital for the establishment of the incompatible interaction [26,27,28]. Moreover, the importance of lipid signalling in grapevine defence against the downy mildew causing agent was highlighted [28,29,30]. During this defence process, one of the key players is JA, a widely studied plant hormone involved in plant responses to several stresses, reviewed in [31]. Upon inoculation with *P. viticola* this signalling molecule is highly accumulated in the tolerant *V. vinifera* crossing hybrid, Regent [27]. Activation of the entire machinery behind the JA synthesis, such as the accumulation of its precursor C18:3, in membrane lipids, concomitant with the up-regulation of several phospholipases A coding genes were also described [28]. These enzymes release C18:3 from membrane lipids, including the plastidial galactolipids, particularly rich in this FA. After release, C18:3 will be available to be further converted in JA. The up-regulation of genes encoding enzymes involved in the JA biosynthetic pathway was also reported in *P. viticola* infected leaves [27].

Despite the importance of lipid and FA modulation in grapevine defence against downy mildew, no information regarding FA desaturases in grapevine is available to date. Thus, with the present study, we intended to fill the gap regarding FA desaturases involvement in grapevine defence against *P. viticola*. A genome-wide characterisation of the FA desaturase gene family was conducted, and the expression of several family members was studied during the first hours of the incompatible grapevine—*P. viticola* interaction. Expression results were correlated with FA modulation occurring in the different lipid classes present in leaves. Our results highlight the important role of FA desaturases in FA modulation induced as a defence response to *P. viticola*.

## 2. Results

### 2.1. Characterisation of Grapevine Fatty Acid Desaturases

#### 2.1.1. Grapevine Fatty Acid Desaturase Gene Family Identification

**Figure 1:**
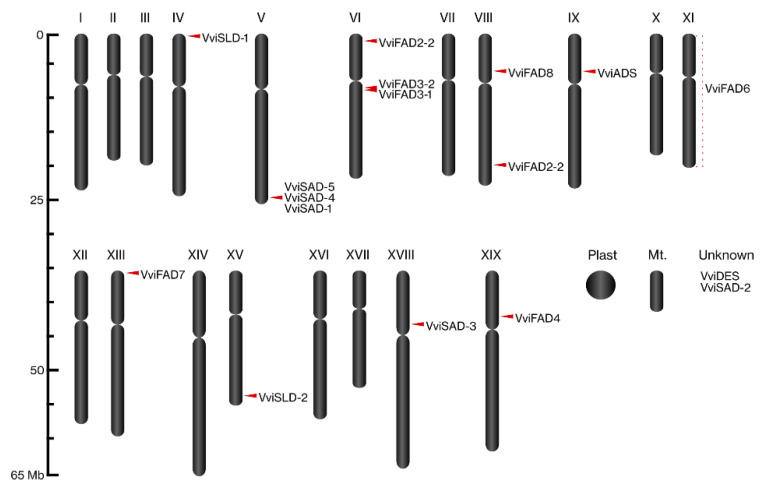
Chromosomal location of *Vitis vinifera* FA desaturases genes. The proposed nomenclature of each *V. vinifera* FA desaturase is shown for each chromosome.

The FA desaturase superfamily members were identified in *Vitis vinifera* through queries using *Arabidopsis thaliana*, *Glycine max*, and *Oryza sativa* protein sequences. A total of 17 genes encoding 17 predicted proteins were identified in *V. vinifera* genome (Supplementary Table S1). The mapping of the identified FA desaturases genes in the 19 grapevine chromosomes shows that they are unevenly distributed in 10 chromosomes, with chromosomes 5 and 6 being the ones where these genes were most represented, containing 3 FA desaturase genes each. Chromosome 8 harboured two genes, and the chromosomes 4, 9, 11, 13, 15, 18, and 19 contained one gene each (Figure 1). The specific location of 2 of the 17 grapevine FA desaturases genes remains unknown (Figure 1).

The exon-intron structure analysis of grapevine FA desaturases genes revealed a discriminating pattern between soluble and membrane-bound FA desaturases. In *V. vinifera*, the number of exons in FA desaturases was between 1 and 10 (Supplementary Table S1). All the grapevine desaturases that had three exons in their gene structure belonged to the *VviSADs*, while the membrane-bound group members had variable numbers of exons. There were only three intronless desaturases in grapevine, *VviSLD-1*, *VviSLD-2*, and *VviFAD4*. *VviADS* is the only gene with five exons, and *VviFAD2* and *VviDES* contain two exons. The desaturase genes with higher numbers of exons were *VviFAD7* and *VviFAD8*, with eight exons, *VviFAD3* with nine exons and *VviFAD6* with ten exons.

#### 2.1.2. Phylogenetic Analysis of Grapevine Fatty Acid Desaturases

**Figure 2:**
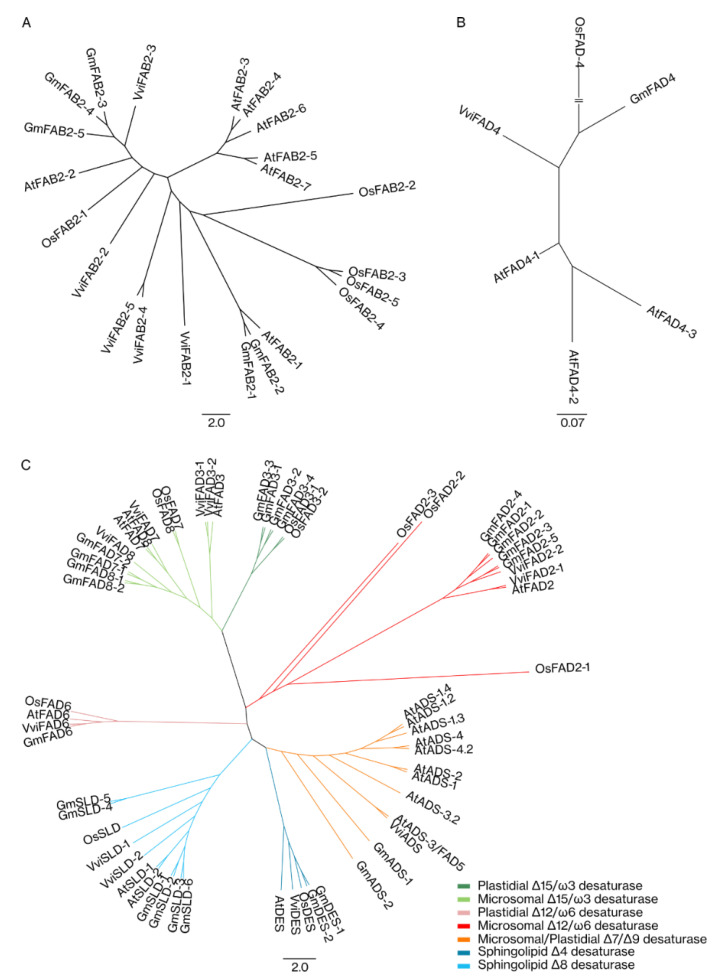
Maximum likelihood phylogenetic tree of the *Vitis vinifera*, *Arabidopsis thaliana*, *Glycine max*, and *Oryza sativa* FA desaturases superfamily. (**A**) Phylogenetic tree of the SAD proteins (**B**) Phylogenetic tree of the Δ3 desaturase proteins; (**C**) Phylogenetic tree of the Δ9 desaturase, Δ12/ω6 desaturase, Δ15/ω3 desaturase, sphingolipids Δ4 and the sphingolipid Δ8 desaturase. In (**B**), the root was truncated with a double dash totalling 0.49 changes per branch length. Scale bar represents the number of estimated changes per branch length.

In order to predict the structure of grapevine FA desaturases superfamily, a phylogenetic analysis of *Vitis vinifera*, *Arabidopsis thaliana*, *Glycine max*, and *Oryza sativa* proteins was performed. With this analysis, we also propose a nomenclature for the members of the grapevine FA desaturases based on sequence identity with Arabidopsis, soy and rice (Figure 2; Figures S1–S3) following the grapevine gene nomenclature method proposed by Grimplet and co-workers [32]. In grapevine, the desaturases were distributed in two major groups, the soluble and the membrane-bound desaturases. These two groups were analysed separately due to the fact that they were evolutionarily unrelated, as soluble desaturases are only present in higher plants [33]. The soluble desaturases were the less represented, being composed of five members (VviSAD-1, VviSAD-2, VviSAD-3, VviSAD-4, and VviSAD-5) (Figure 2A), while membrane-bound desaturases were the most represented (Figure 2B,C). This group contained the acyl-lipid desaturases and was subdivided into several sub-groups: the Δ3 desaturase, Δ9 desaturases, Δ12/ω6 desaturases, Δ15/ω3 desaturases, sphingolipids Δ4, and sphingolipid Δ8 desaturase. Although the Δ3 desaturase subfamily belongs to the membrane-bound group, these desaturases evolved independently from the other members and, because of that, were analysed separately from the remaining group [19]. In grapevine, only one member of the Δ3 desaturase group (VviFAD4) was identified (Figure 2B). The remaining membrane-bound desaturases were analysed simultaneously (Figure 2C). The Δ9 desaturases sub-group was composed of one member (VviADS); the Δ12/ω6 desaturases were comprised of three members (VviFAD2-1, VviFAD2-2, and VviFAD6); the Δ15/ω3 desaturases were the most represented sub-group containing four members (VviFAD3-1, VviFAD3-2, VviFAD7, and VviFAD8); sphingolipids Δ4 desaturases had one member (VviDES), and the sphingolipids Δ8 desaturase sub-group was composed of two members (VviSLD-1 and VviSLD-2).

#### 2.1.3. Identification of *Cis*-Elements of Grapevine *FA Desaturases* Genes

An enrichment analysis of *cis*-regulatory elements in *FA desaturase* genes promoters was conducted for a deeper comprehension of the transcription regulation and gene function of grapevine *FA desaturases* (Supplementary Table S2). The conservative promoters elements, CAAT- and TATA- boxes, were found in all *FA desaturases* genes sequences. Several *cis*-acting elements related to plant response to phytohormones, stress, development and growth were identified. Regarding phytohormone responses, *cis*-elements related to abs*cis*ic acid (ABRE) were found. The most represented were those found in 15 *FA desaturases* promoters, methyl jasmonate (CGTCA- and TGACG-motfis) in 7 *FA desaturases* promoters, salicylic acid (TCA-element) in 11 *FA desaturases*, auxin (TGA-element and AUXRR-core) in 4 *FA desaturases* promoters, and gibberellin (GARE-motif, TATC-box and P-box) in 6 grapevine *FA desaturases* promoters. *Cis*-elements associated with stress and defence responsiveness (TC-rich repeats) were observed in 7 *FA desaturases* promoters, wound responsive element (WUN-motif) was observed in 1 *FA desaturase* promoters, anaerobic induction (ARE) was found in 15 *FA desaturases* promoters, light response (GATA-, GA-, TCT-, AT1-, ATCT- GT1- and TCC-motifs, AE-, I-, G-, and Gap-boxes, ACE, MRE, LAMP element, Chs-CMA1a and box4) in 16 *FA desaturases*, and 5 grapevine *FA desaturases* showed the presence of *cis*-elements associated with responsiveness to low temperature (LTR). Finally, some *cis*. Elements involved in plant development and growth (CAT-box) was found in nine *FA desaturases* promoters, whereas the *cis*-elements related with seed regulation (RY-element) and expression regulation element (GCN4_motif) were observed in one and two grapevine *FA desaturases* promoters, respectively. The MYB binding responses elements (MBS and MBSI) were also identified in the promoter regions of grapevine *FA desaturases*.

#### 2.1.4. Protein Structure and Domain Analysis

Grapevine FA desaturases size varies between 307 and 456 amino acids, with an average length of approximately 397 amino acids (Supplementary Table S1). The predicted molecular weight of these proteins varied from 34.52 kDa and 51.97 kDa, and the isoelectric point ranged from 6.14 to 9.51 (Supplementary Table S1).

**Figure 3:**
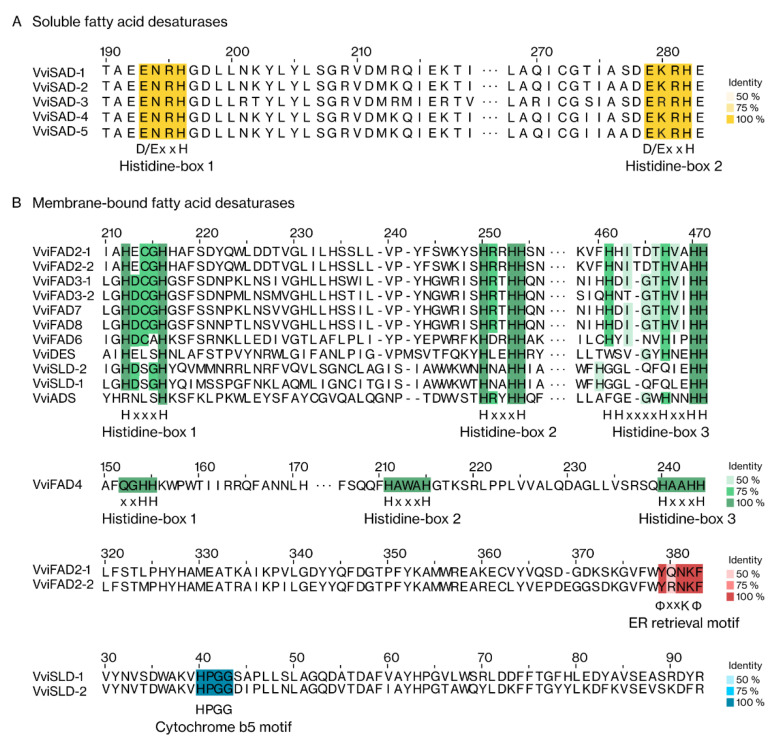
Multiple alignments of the two grapevine FA desaturases groups representing the consensus and conserved motifs. Protein sequences were aligned for each FA desaturase group separately, applying the MAFFT tool. The consensus motifs are shown in shadow boxes according to percentage identity. (**A**) soluble fatty acid desaturases; (**B**) membrane-bound desaturases.

**Figure 4:**
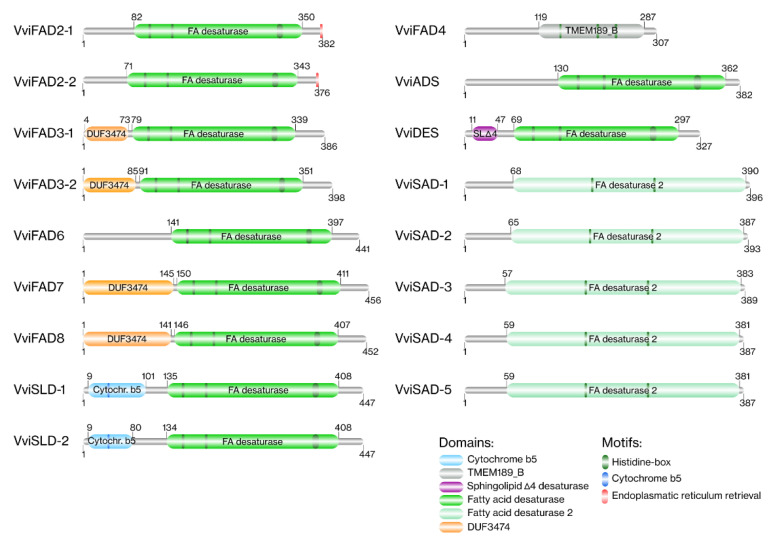
Protein domains and motifs of the grapevine FA desaturases.

The identification of motifs and domains is an important aspect in protein characterisation, allowing their classification and functional annotation. Each group of FA desaturases has highly conserved domains and motifs, which are distinctive features of each family (Figure 3 and Figure 4; Supplementary Tables S3 and S4). To identify the consensus and conserved motifs in each grapevine FA desaturase protein, a multiple sequence alignment was performed (Figure 3). In grapevine, the soluble desaturases proteins had two highly conserved histidine-boxes (D/ExxH), whereas the membrane-bound desaturases proteins had three histidine-boxes in their sequences (HxxxH, HxxxH, and HHxxxxHxxHH). Despite showing three histidine-boxes, the motifs in VviFAD4 differ from those present in the remaining membrane-bound desaturases. VviFAD2-1 and VviFAD2-2 had a C-terminal ER motif, YQNKF and YRNKF, respectively. A highly conversed cytochrome b5 motif (HPGG) at N-terminus was also observed in both VviSLDs.

Concerning domains, a prediction regarding all grapevine FA desaturases was performed (Figure 4; Supplementary Table S4) using the Pfam database. All the VviSAD members had a FA desaturase 2 domain in their protein sequence. The membrane-bound desaturases, with the exception of VviFAD4, had a FA desaturase domain. In VviFAD4, a TMEM189_B domain was identified. In VviFAD3, VviFAD7 and VviFAD8 proteins, a domain of unknown function (DUF3474) at the N-terminus was observed. The sphingolipids’ desaturases also had specific domains. An N-terminal cytochrome b5 domain and a sphingolipid Δ4 desaturase domain were found in VviSLD and VviDES sequences, respectively.

#### 2.1.5. Grapevine FA Desaturases Subcellular Targeting Prediction

Each FA desaturase acts in a specific cellular location. We further analysed the predicted subcellular location for all grapevine FA desaturases. Our results showed that all the soluble desaturases (VviSAD) were predicted to localise in the chloroplast. Regarding the membrane-bound desaturases, as expected, they were not restricted to the chloroplast, being some members located in the ER. The VviFAD4, VviFAD5, VviFAD6, VviFAD7, VviFAD8, and VviADS proteins were predicted to be located in chloroplast, whereas the VviFAD2, VviFAD3, VviSLD, and VviDES were identified as ER enzymes (Supplementary Tables S1 and S5).

### 2.2. Fatty Acid Desaturases Gene Expression Correlates with Changes in the Fatty Acid Profiles upon Pathogen Inoculation

Our previous works suggested that lipids and FA are intimately related to the establishment of an incompatible interaction of the tolerant *V. vinifera* cultivar Regent with *P. viticola* [28]. The progressive FA desaturation of membrane glycerolipids upon inoculation with the downy mildew agent is crucial. Taking this into account, the genes encoding the FA desaturases involved in the desaturation of plastidial glycerolipids (monogalactosyldiacylglycerol (MGDG), digalactosyldiacylglycerol (DGDG) and phosphatidylglycerol (PG)) and extraplastidial glycerolipids (phosphatidylcholine (PC) and phosphatidylethanolamine (PE)) were selected for expression analyses, in order to elucidate their involvement in the establishment of the incompatible interaction.

**Figure 5:**
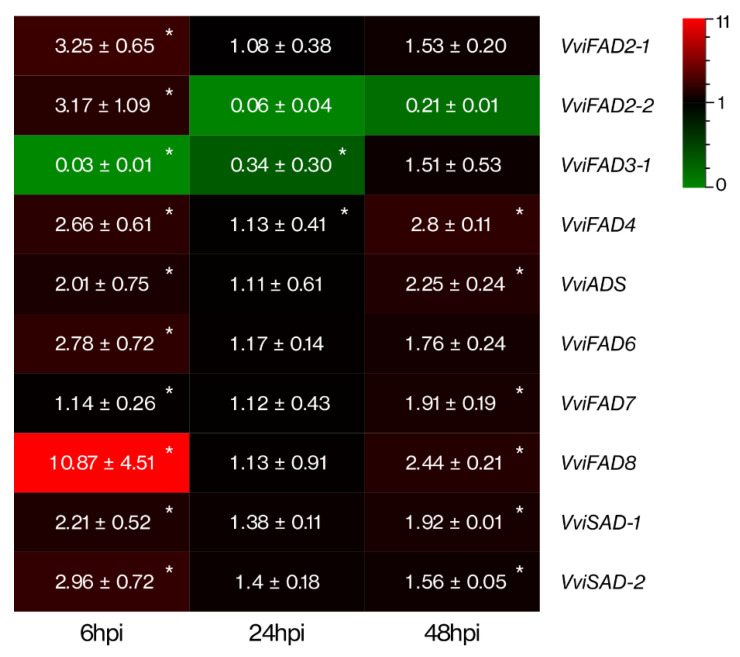
Expression analysis of FA desaturases genes of *V. vinifera* cv. Regent leaves upon inoculation with *P. viticola*. The gene transcript’s fold-change relative to controls, at each time point (6, 24 and 48 hpi), are represented for: *VviFAD2-1*; *VviFAD2-2*; *VviFAD3-1*; *VviFAD4*; *VviADS*; *VviFAD6*; *VviFAD7*; *VviFAD8*; *VviSAD-1*; *VviSAD-2*. Fold-change values are relative to expression in mock-inoculated leaves. Asterisks indicate significant differences (*p* < 0.05).

The expression profiles of *VviFAD2-1*, *VviFAD2-2*, *VviFAD3-1*, *VviFAD3-2*, *VviFAD4*, *VviADS*, *VviFAD6*, *VviFAD7*, *VviFAD8*, *VviSAD2-1*, and *VviSAD2-2* genes were analysed by qPCR in *V. vinifera* cv. Regent leaves at 6, 24 and 48 hours post-inoculation (hpi) with *P. viticola*. The expression of the majority of the selected genes was up-regulated, mostly at 6 hpi (Figure 5). The *VviFAD3-1* was the only gene down-regulated at 6 and 24 hpi, but to a small extent and returning to control levels at 48 hpi. The remaining FA desaturases genes presented an up-regulation at 6 hpi, decreasing their expression at 24 hpi to basal levels. Out of the 10 selected genes, 6 (*VviFAD4*, *VviADS*, *VviFAD7*, *VviFAD8*, *VviSAD2-1*, and *VviSAD2-2*) had their expression increased at 48 hours after the pathogen challenge. Among them, the expression levels of *VviFAD8*, *VviSAD2-1*, and *VviSAD2-2* were higher at 6 hpi, whereas the *VviFAD4*, *VviADS*, and *VviFAD7* showed higher expression levels at 48 hpi. *VviFAD4* was the only gene presenting an up-regulation at all time points. The expression *VviFAD3-2* was not detected due to low transcript abundance.

**Figure 6:**
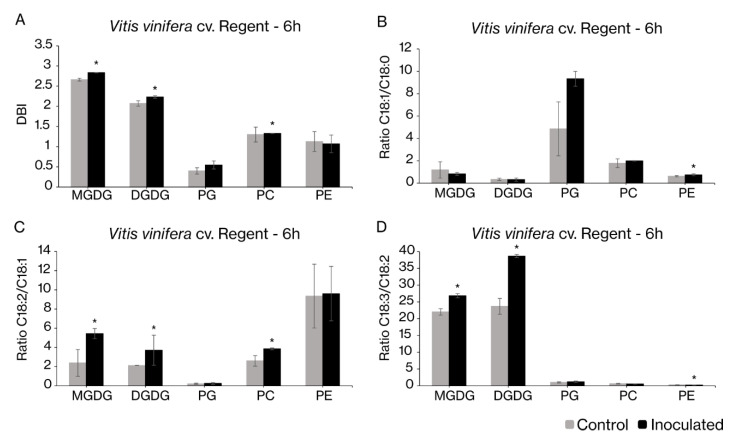
Fatty acid-related parameters of major leaf lipid classes of *V. vinifera* cv. Regent mock control and inoculated leaves with *P. viticola* at 6 h. (**A**) Double bound index (DBI); (**B**) Ratio between C18:1 and C18:0; (**C**) Ratio between C18:2 and C18:1; (**D**) Ratio between C18:3 and C18:2. Values correspond to average values ± standard error, *n* = 5; Asterisks indicate significant differences (*p* < 0.05).

Taking into account that the bigger changes in desaturase gene expression were observed 6 hpi, the changes in the FA composition of the major lipid classes (chloroplast galactolipids, MGDG and DGDG, and phospholipid PG) and the extraplastidial lipids (PC and PE) were evaluated at this time-point. After the pathogen challenge, most lipids showed a modulation in their FA composition (Figure 6). PC, MGDG and DGDG exhibited an increase in their double bound index (DBI) (Figure 6A). This modification is the result of the increase in unsaturated FAs, mainly PUFAs, which are well represented by the FA ratios (Figure 6B–D). In order to compare the changes in FA profiles with the alterations of expression of the different desaturases, the ratios between specific FAs were calculated. The main differences were related to the PUFAs, C18:2 and C18:3, where the increase in the ratio between C18:2 and C18:1 in MGDG, DGDG and PC (Figure 6C) and an increase in the ratio between C18:3 and C18:2 in MGDG and DGDG (Figure 6D) were observed. Despite the tendency observed for PG, in agreement with changes observed for the FA ratios of the other plastidial lipids, no significant alteration was observed (Figure 6). In PE, an increase in the ratio regarding monosaturated (C18:1) and saturated (C18:0) FAs (Figure 6B) and a decrease in the ratio between C18:3 and C18:2 were also observed (Figure 6D).

## 3. Discussion

### 3.1. Characterisation of Fatty Acid Desaturases in Grapevine

The FA desaturation is one of the basic processes in plant metabolism, acting on plant homeostasis, growth, reserve, and defence. Our analysis showed that the grapevine FA desaturases are distributed into two main groups, soluble, and membrane-bound FA desaturases, as reviewed in [34]. The first group was the less represented, comprising about a quarter of FA desaturases. In grapevine, five soluble desaturases were identified in accordance with the SAD proportion previously observed in Arabidopsis and peanut, comprising seven [35] and three [14] SAD members, respectively. The VviSAD proteins were predicted to be located in chloroplast, where they may participate in the desaturation of C18:0 forming C18:1-ACP. As expected, they had two histidine motifs and a specific domain, which were involved in binding the diiron complex used for the catalysis of the double bond introduction at the Δ9 position of C18:0, forming C18:1 [36].

On the other hand, the membrane-bound desaturases compose the larger group with 12 predicted members in grapevine. These desaturases had a FA desaturase domain, except for VviFAD4 and three highly conserved histidine-boxes. Both FA desaturase domain [10] and histidine-boxes [37] are found in membrane-bound desaturase that are able to catalyse the insertion of a double bond in the acyl chain, essential for the catalytic activity of the enzymes, by forming a cluster of two metal ions in the catalytic complex [37]. In grapevine, only one FAD4 was identified, in contrast to Arabidopsis, which has three FAD4 members [19], and peanuts, which has two members [14]. The VviFAD4 was predicted to be a chloroplastidial desaturase responsible for the synthesis of C16:1 *t* from C16:0 in PG. Contrarily to the remaining membrane-bound enzymes, VviFAD4 did not have a FA desaturase domain, having a TMEM189_B domain instead, a transmembrane domain also present in Arabidopsis FAD4 [19]. In spite of presenting three histidine-boxes, the VviFAD4 histidine motif (QGHH) differed from those present in membrane-bound desaturases, probably due to an evolutionary process that occurred independently from the other proteins [19]. Three Δ12/ω6 desaturases were recognised in *V. vinifera*: two FAD2 members (VviFAD2-1, VviFAD2-2) and one FAD6 (VviFAD6). The same number of FAD6 members was also found in Arabidopsis and cotton, with only one member each [14,20]. Regarding FAD2, Arabidopsis has only one member [21], whereas peanut has three FAD2 members [14]. They are responsible for the synthesis of C18:2, by adding a second double bond in C18:1 esterified to phospho- or glycolipids. VviFAD2 was predicted as a microsomal desaturase with a C-terminal ER-retrieval motif in their protein sequence, as described in [8]. Whereas, VviFAD2 acts on ER membranes, VviFAD6 is predicted to be a chloroplast enzyme involved in the synthesis of C18:2, as defined for their Arabidopsis orthologous [20,21]. The Δ15/ω3 subgroup harbours the members VviFAD3-1, VviFAD3-2, VviFAD7 and VviFAD8 associated with the synthesis of C18:3. Arabidopsis had fewer members belonging to this subgroup, possessing only one member of each desaturase [22,23], whereas peanuts have three FAD3, two FAD7, and no FAD8 characterised so far [14]. In these proteins, a domain of unknown function (DUF3474) at the N-terminus was observed. Despite not having a characterised function, this domain is found in bacteria and eukaryotic organisms, being associated with the FA desaturase domain. This subgroup is in charge of the synthesis of C18:3 by adding a third double bond to C18:2 esterified to phospho- or glycolipids. While VviFAD3 is predicted to be a microsomal desaturase [22], which has the ER-membrane as a target, the VviFAD7 and VviFAD8 have been predicted to have the chloroplast lipid membrane as a target [23]. In grapevine, the subgroup of acyl-lipid Δ9 desaturases was comprised of only one member, the VviADS, similarly to peanuts [14], whereas in Arabidopsis, nine ADS members have been described [38]. The grapevine ADS presented high identity with AtADS3 (also called AtFAD5). AtADS3, the first well-characterised enzyme of the ADS group, is involved in the synthesis of C16:1 *cis* from C16:0 in MGDG [18,39]. C16:1 *cis* is further desaturated into C16:2 and C16:3, the latter comprising a significant percentage of galactolipid FAs in the so-called 16:3 plants, such as Arabidopsis but is absent in 18:3 plants like grapevine [39,40]. Despite having a high identity to the AtADS3, it is likely that VviADS may be responsible for the production of C18:1 in plastidial lipids instead of C16:1, as it was observed for ADS2, which has been shown to catalyse the desaturation of C16:0 and C18:0 esterified to PG or MGDG [41]. In sphingolipids, FA desaturation is carried out by acyl-lipid Δ4 and Δ8 sphingolipids desaturases, the SLD [24] and DES [25]. In aAabidopsis, two SLDs [24] and one member of DES [25] have been identified. In peanuts, four SLDs were found, and one DES was characterised [14]. Our results demonstrated the presence of two SLDs and one DES in the grapevine genome. These two sphingolipid desaturases, besides sharing a FA desaturase domain with the remaining membrane-bound desaturases, present specific conserved regions. Both VviSLD had a cytochrome b5 motif as well as a cytochrome b5 domain at the N-terminus. These conserved regions are found in other plant acyl-lipid SLD desaturases that use the cytochrome b5 as an electron donor in the desaturation process [24]. DES, which evolved independently from the SLD, do not contain a cytochrome b5 [25]. They have a small domain associated with their FA desaturase domain, called the sphingolipid Δ4 desaturase domain [25]. This domain was also detected in the protein sequence of VviDES.

### 3.2. Fatty Acid Desaturases in Grapevine Defence

Lipid signalling has been emphasised in grapevine defence mechanisms against *P. viticola* [26,27,28]. As a result, both lipid and FA suffer modifications, where the chloroplast membrane lipids, MGDG and DGDG, were the most affected. These lipid classes are accumulated, and their content in C18:3 is increased [28]. Upon pathogen inoculation, Regent galactolipids showed an increase in their C18:2 and C18:3 content. The accumulation of these PUFAs translates into a higher DBI, which is associated with a more fluid and permeable lipid membrane. The biological membranes in eukaryotic cells are composed of a lipid bilayer that separates the interior of the cell from the outside environment, protecting the cell interior [42]. Besides the plasma membrane, each cell organelle also has its own membranes with specific lipid composition [42]. In response to stressful conditions, cell membranes can adjust their degree of FA desaturation in order to modify their membrane properties, becoming more or less fluid in response to salinity, humidity or temperature [42]. In our previous work, we have highlighted that the grapevine genotype Regent adjusted its proportion of PUFAs, upon *P. viticola* inoculation, increasing the DBI and promoting a more fluid chloroplast membrane [28]. In that work, we suggest that after pathogen challenge, the increase in chloroplasts’ membrane fluidity may be crucial to avoid any damage in the photosynthetic machinery, which represents inevitable effects on the energy transduction pathways and primary productivity [28].

To determine the participation of the grapevine FA desaturases in grapevine defence against downy mildew, we conducted an expression analysis and found that the transcript levels of the majority of genes increased as soon as 6 h upon pathogen challenge. At 6 hpi, most of the genes had their expression up-regulated. The following time-point (24 h) showed a decrease, almost to the basal levels and the latest time-point (48 h) showed a small increase in the expression of some genes. The gene expression oscillation between time-points could be explained by a bimodal regulation. In this type of defence response, a quick and temporary initial signal is detected, followed by a later one, or more signals, of lower amplitude, but more lasting [43]. The bimodal regulation has been associated with Pattern-Triggered Immunity and Effector Triggered Immunity, activated by [Ca^2+^]cyt and reactive oxygen species (ROS) bursts [43].

Both grapevine FAD2 isoforms, *VviFAD2-1* and *VviFAD2-2* showed homology with Arabidopsis Δ12/ω6, which encodes ER-located enzymes responsible for the formation of C18:2 in membrane lipids [21]. Their expression was up-regulated at 6 hpi, decreasing in the following time-points. These results are in accordance with our lipid analyses, where an increase in the C18:2/C18:1 ratio in PC was observed after pathogen interaction. In contrast, *VviFAD3-1* was down-regulated in response to *P. viticola* at 6 and 24 h, increasing at 48 h. The encoded enzyme desaturates C18:2 in C18:3 in PC and could also impact the FA composition of other extraplastidial lipids by acyl editing mechanisms. The down-regulation of *VviFAD3-1* was connected to the decrease in the C18:3/C18:2 ratio in occurring PE and the similar tendency observed in PC.

At the chloroplast level, all the desaturases coding genes were up-regulated. Both *VviSAD* and *VviADS* have their expression increased at 6 and 48 hpi. These genes encode proteins homologous to the AtSAD and AtADS proteins that have been described as desaturases involved in the C18:1 synthesis from C18:0. SAD acts on ACP-bound FA, forming C18:1-ACP. The ADS enzymes act on acyl-lipids. Since ADS is predicted to be located in the chloroplast and being grapevine an 18:3 plant, it is likely that this desaturase contributes to the formation of C18:1 in plastidial glycerolipids (rather than contributing to C16:1 synthesis that occurs in 16:3 plants). Indeed, although not statistically significant, a tendency for the increase in the ratio C18:1/18:0 content in PG is shown. C18:1-ACP can also be used in the chloroplast as a substrate of lipids of this organelle or exported to the ER and incorporated in extraplastidial lipid classes. The increased levels of expression in *VviSAD-1* and *VviSAD-2* are in agreement with our data, where an increase in C18:1/18:0 in PE was observed, and a similar tendency was also seen in PC. Besides that, an accumulation of galactolipids in response to *P. viticola* has previously been observed [28]. The activation of *VviSAD* genes could be linked to this observation by the fact that they could be responding to the high demand for C18:1-ACP, used for galactolipid synthesis. Additionally, a higher expression of these genes could also be related to an increase in plant resistance to downy mildew. In other plants, a higher expression of SAD induced resistance against pathogens, including powdery mildew [44,45], whereas *SAD* (*ssi/fab2*) mutants, lacking desaturase activity (C18:0 > C18:1), demonstrate high susceptibility to *Botrytis cinerea* [46]. Another desaturase analysed was *VviFAD4*, which has homology with *AtFAD4*. This desaturase is PG specific and is responsible for the desaturation of C16:0 in C16:1 *trans* [19]. In our previous work, the analysis of the leaf FA pool revealed an accumulation of this FA in Regent at the first hours of *P. viticola* challenge. This was linked to a tendency to an increase in the relative amounts of PG [28], which can also be related to the up-regulation of *VviFAD4* here observed.

The following grapevine desaturases analysed are related to the most significant modifications observed in grapevine leaf lipid metabolism. The *FAD6*, *FAD7*, and *FAD8*, genes encode the enzymes responsible for PUFAs production in chloroplast lipids. The production of C18:2 is performed by FAD6, while C18:3 synthesis is carried out by FAD7 and FAD8. Our data showed an increase in *VviFAD6*, *VviFAD7* and *VviFAD8* expression at 6 hpi, where an increase in the ratios C18:2/C18:1 and C18:3/C18:2 in galactolipids was also observed. Chloroplast PUFAs could actively participle in plant defence responses to pathogen attacks by serving as substrate for signalling molecules, acting themselves as signalling molecules or by changing the membrane properties [31]. High C18:2 levels were already shown to enhance the resistance of avocado to *Colletotrichum gloeosporioides* attack [47]. In contrast, the Arabidopsis double mutant *fad2 fad6* presents its photosynthesis impairment as a consequence of the lower content in PUFAs [48]. This evidence gives strength to our previous hypothesis that the increase in PUFAs’ content is a protective strategy to prevent the membrane disruption protecting the photosynthetic machinery by turning the chloroplast membrane more fluid and permeable [28]. C18:3 was also shown to be released from chloroplast membrane lipids and act either directly as signalling molecule modulating a myriad of signals or indirectly by acting in signal mechanisms as a substrate for the biosynthesis of other signalling molecules [31]. Firstly, PUFAs can act directly, inducing NADPH oxidase activity and subsequently modulating ROS production [49]. ROS production is capable of trigger several defence responses, and hypersensitive response during the Effector Triggered Immunity, reviewed in [49]. The desaturases yielding C18:3 are intimately related to this phenomenon. Arabidopsis mutants *fad7* and *fad8* have their aptitude for producing ROS compromised, demonstrating extreme susceptibility to bacterial pathogens [50]. Additionally, we have previously observed a ROS burst in Regent at 6 hpi with *P. viticola* [29], which allied to the oscillating gene expression, between 6–48 hpi, suggests a possible bimodal regulation in grapevine as response to downy mildew. Secondly, PUFAs can act indirectly, serving as substrate for oxilipins biosynthesis, such as JA [31]. This signalling molecule has been recognised by its important function in plant defence response either to abiotic either to biotic stress [31]. In grapevine, JA has also a significant role in response to *P. viticola*, being accumulated in the first hours of infection [27]. The importance of FA desaturases in JA formation as well as in resistance to pathogen has been shown. In *fad3*, *fad7*, and *fad8* Arabidopsis triple mutants, the lack of desaturase activity led to the inability to accumulate jasmonates, making them highly prone to *Pythium mastophorum* [51].

### 3.3. Fatty Acid Modulation in Grapevine-Plasmopara Viticola Interaction

Lipid signalling has been shown to be a key component of grapevine defence mechanisms against *P. viticola*. As a result, both lipids and FA suffer modifications, where the chloroplast membrane lipids, MGDG and DGDG, are the most affected. These lipid classes accumulate, and their content in C18:3 increases [28]. Besides these alterations, lipid hydrolysis [28], lipid peroxidation [26], and further activating of the JA biosynthesis pathway occur [27]. In the present study, we elucidated how lipid signalling was initiated through a comprehensive overview of FA desaturation events in grapevine defence against *P. viticola*. After the pathogen challenge, the most affected lipid classes were those concerning chloroplast membranes, MGDG and DGDG, reinforcing the importance of galactolipids metabolism in grapevine defence mechanisms. These galactolipids have been described in the establishment of the defence responses by regulating systemic acquired resistance (SAR) in distinct pathways [52] and by supplying the substrates for oxylipins synthesis [31]. DGDG contributes to salicylic acid and nitric oxide biosynthesis, regulating SAR, whereas MGDG has effects downstream of nitric oxide, inducing azelaic acid biosynthesis and promoting SAR [52]. Oxylipins are potent secondary signal molecules that act in plant defence indirectly, amplifying the initial stimulus received by the plant and directly as anti-microbial compounds. They are originated from PUFAs that suffer a series of oxidative processes. Jasmonic acid, one of the most studied oxylipins, is associated with several physiological, developmental and defence responses [31]. Upon pathogen inoculation, Regent galactolipids showed an increase in their C18:3/C18:2 and C18:2/C18:1 ratios. The accumulation of these PUFAs, C18:2 and C18:3, results in a higher DBI, which is related to changes in membrane properties. As a response to pathogen attack, the chloroplast membranes will become more fluid and permeable. A higher fluidity and plasticity will prevent membrane disruption, preserving the photosynthetic apparatus and avoiding any perturbation in photosynthesis [28]. It is also possible to hypothesise that a more permeable membrane could have an impact on the molecular exchange, such as ions and oxylipins’ substrates, facilitating their transport between the chloroplast stroma and the cytosol. Besides the physical changes, a higher amount of C18:3 means a higher bio-availability of this FA to be channelled to the JA biosynthesis pathway [27].

## 4. Materials and Methods

### 4.1. Plant Material

The experimental procedures were conducted in the grapevine cultivar *V. vinifera* cv. Regent. This cultivar is a hybrid, bred at Julius Kuhn Institute (JKI, Germany), with a high tolerance to *P. viticola* loci 3.1 (RPV3.1), presenting a high degree of tolerance to downy and powdery mildew. Woody shoots were grown according to previously optimised conditions [53]. *Plasmopara viticola* sporangia were collected from symptomatic leaves from field-infected plants and were collected as previously described [53]. The *P. viticola* sporangia vitality was confirmed by microscopy [54]. The abaxial leaf surface was sprayed with an aqueous suspension containing 10^4^ sporangia mL^−1^, while controls were sprayed with water (mock inoculations). At each time-point, 6, 24, and 48 h, both mock and inoculated leaves were harvested and immediately frozen in liquid nitrogen and stored at −80 °C, as described in [28]. Five independent biological replicates were collected for each condition (inoculated and mock-inoculated).

### 4.2. Lipid Analysis

Ground leaves were boiled in water for 5 min to inactivate lipolytic enzymes. A solution of chloroform/methanol/water (1:1:1, *v*/*v*/*v*) was used to extract lipophilic compounds, as previously described in [28]. Lipid classes, at 6 hpi, were separated by a thin layer chromatography using the following mobile phase: chloroform/methanol/acetone/acetic acid/water (100/20/40/20/8, *v*/*v*/*v*/*v*/*v*), on silica plates (G-60, Merck, VWR, Radnor, PA, USA). Lipid bands were visualised under UV light, using a solution of 0.01% primuline in 80% acetone (*v*/*v*), being posteriorly scraped off. The trans-methylation of FA was carried out using a solution of methanol:sulfuric acid (97.5:2.5, *v*/*v*) for 1 h at 70 °C, in order to generate FA methyl esters (FAMEs). FAMEs were collected in the organic phase upon adding petroleum ether:ultrapure water (3:2, *v*/*v*). The quantitative analysis of FAMEs was accomplished using gas chromatography (430 Gas Chromatograph, Varian), according to previously optimised conditions [28]. Heptadecanoic acid (C17:0) was used as an internal standard the double bound index (DBI) was calculated as follows:

DBI = (% monodienoic acids) + 2 (% dienoic acids) + 3 (% trienoic acids)/100

### 4.3. Characterisation of the Grapevine Fatty Acid Desaturases

#### 4.3.1. Identification and Retrieval of Grapevine FA Desaturases Sequences

The predicted grapevine FA desaturases genes and protein sequences were identified in the NCBI BLAST tool (https://blast.ncbi.nlm.nih.gov/Blast.cgi) (accessed on November 2020) [55] using *Arabidopsis thaliana*, *Oryza sativa*, and *Glycine max* FA desaturases protein sequences as a query for blast searches. The following databases were used to search the protein sequences of each FA desaturases, used as a query: TAIR (https://arabidopsis.org) (accessed on January 2020) [56], RGAP (https://rice.plantbiology.msu.edu) (accessed on January 2020) [57] and Phytozome v12.1 (www.phytozome.net) (accessed on January 2020). The identified sequences were submitted for a Hidden Markov Model search, using the HMMER software [58] and the Pfam database [59], for the FA desaturase domain, type 1 or type 2 (PF00487 and PF03405, respectively), with a cut-off e-value of 0.01. The putative grapevine FA desaturases sequences were further confirmed on CRIBI database (V2 annotation) (http://genomes.cribi.unipd.it/grape/) (accessed on November 2020) [60].

#### 4.3.2. Cis-Element Analysis for Grapevine FA Desaturases GENE Promoter

The database Phytozome v12.1 (www.phytozome.net) (accessed on May 2021) was used to search the promoter sequence of 2000 bps upstream of each *FA desaturase* gene coding regions. The *cis*-elements analysis was conducted using the PlantCARE database (http://bioinformatics.psb.ugent.be/webtools/plantcare/html/) (accessed on May 2021) [61].

#### 4.3.3. Domain Structure Analysis, Sequence Properties, Subcellular Location Prediction and Chromosomal Location

The database Pfam (http://pfam.xfam.org/) (accessed on November 2020) was used to predict the domain and clan. Isoelectric point and molecular weight were determined using the ProtParam tool from ExPASy (http://web.expasy.org/protparam/) (accessed on November 2020) [62]. Subcellular location prevision was conducted using Predotar (https://urgi.versailles.inra.fr/predotar/) (accessed on November 2020) [63], Localizer (http://localizer.csiro.au/) [64], and TargetP (http://www.cbs.dtu.dk/services/TargetP/) (accessed on November 2020) [65]. The chromosomal location of the FA desaturases’ genes in *V. vinifera* chromosomes were mapped using the Map Viewer tool from NCBI (http://www.ncbi.nlm.nih.gov/mapview/) (accessed on November 2020). All the molecular predictions were manually curated and compiled.

#### 4.3.4. Phylogenetic Analysis

The alignment of the FA desaturases’ protein sequences from *V. vinifera*, *A. thaliana*, *O. sativa*, and *G. max* was performed using the MAFFT software, with the E-INS-I option v7 (http://mafft.cbrc.jp/alignment/software/) [66]. The percentage identity was done with Jalview software (http://www.jalview.org/) [67]. Maximum-likelihood phylogenetic analysis was performed with RAxML-HPC v8, on the CIPRES Science Gateway (https://www.phylo.org) (accessed on January 2021) [68], according to previously optimised conditions [28]. The phylogenetic trees were viewed and edited on FIGTree (http://tree.bio.ed.ac.uk/software/Figtree/) and on Inkscape (http://www.inkscape.org/), respectively.

### 4.4. Expression Analysis

Total RNA was extracted from *V. vinifera* cv. Regent, inoculated and mock-inoculated, frozen leaves using the Spectrum™ Plant Total RNA Kit (Sigma-Aldrich, USA) with minor modifications. Residual genomic DNA was hydrolysed using On-Column DNase I Digestion (Sigma-Aldrich, Saint Louis, MO, USA), according to the manufacturer.

RNA integrity was analysed by agarose gel electrophoresis while concentration and quality (260/280 and 260/230 ratios) were performed using a NanoDrop-1000 spectrophotometer (Thermo Scientific). All samples were analysed by a quantitative real-time Polymerase Chain Reaction (qPCR) of a reference gene, *Elongation Factor 1-alpha* (*EF1α*), on crude RNA in order to detect DNA contamination. Complementary DNA was synthesised from 2.5 μg of total RNA using RevertAid^®^H Minus Reverse Transcriptase (Fermentas, Ontario, Canada) anchored with Oligo(dT)23 primer (Fermentas, Burlington, ON, Canada) as previously described [28]. The qPCR experiments were performed according to previously optimised conditions [28]. Each reaction contained 2.5 mM MgCl2, and 2 μM of each primer were used in 10 μL volume reactions, with 1 μL of cDNA as template. A control without cDNA template was included in each set of reactions. Primer sequences are provided in (Supplementary Table S6). In order to confirm the existence of non-specific PCR products and the single product amplification, a dissociation curve analysis was performed (Figure S4). For each sample, three biological replicates and two technical replicates were used. Gene expression (fold change) was calculated as described in [69]. Ubiquitin-conjugating enzyme (UBQ) and EF1α coding genes were used for expression data normalisation as described in [70].

### 4.5. Statistical Analysis

Statistical analyses of all data were carried out by the Mann–Whitney U test using IBM^®^ SPSS^®^ Statistics software (version 23.0; SPSS Inc., Chicago, IL, USA). The analysis was based on non-parametric tests due to the lack of data normality and homogeneity of variances. Results with a *p*-value < 0.05 were considered statistically significant.

## 5. Conclusions

**Figure 7:**
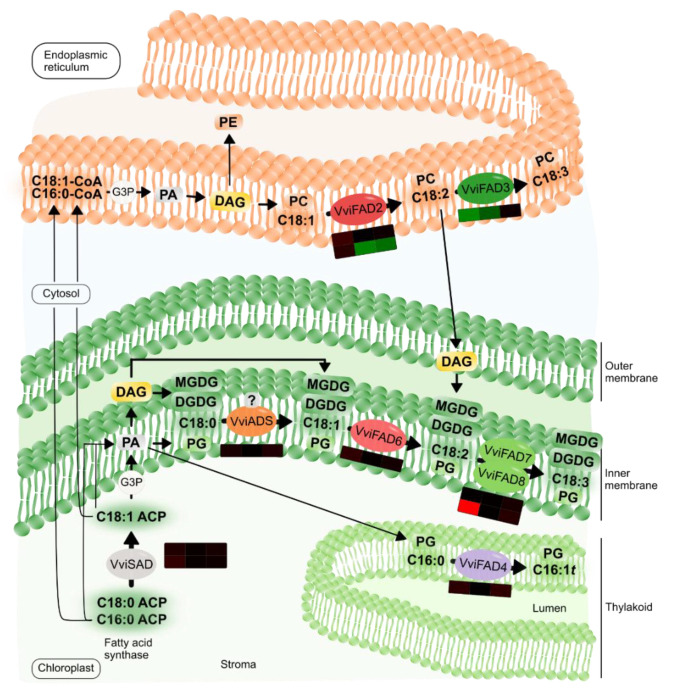
Lipid and FA metabolism, catalysed by the action of FA desaturases, in *V. vinifera* cv. Regent leaves upon inoculation with *P. viticola*. The gene expression regarding each grapevine desaturase encoding gene is represented by the respective heatmap as presented in (Figure 5). Abbreviations: acyl carrier protein (ACP); palmitic acid (C16:1); palmitoleic acid (C16:1); *trans*-9-hexadecenoic acid (C16:1 *t*); oleic acid (C18:1); linoleic acid (C18:2); α-linolenic acid (C18:3); Cytidine diphosphate diacylglycerol (CDP-DAG); coenzyme A (CoA); diacylglycerol (DAG); di–galactosyldiacylglycerol (DGDG); glyceraldehyde 3-phosphate (G3P); mono–galactosyldiacylglycerol (MGDG); phosphatidic acid (PA); phosphatidylcholine (PC); phosphatidylethanolamine (PE); phosphatidylglycerol (PG).

Our work brings novel insights into the participation of lipid metabolism in the establishment of incompatible interaction between grapevine and *P. viticola*. We presented evidence of the crucial role of FA desaturation in the grapevine defence response to downy mildew (Figure 7). The FA desaturases act actively in chloroplast lipids, leading to the formation of PUFAs (Figure 7). This action plays a dual role by protecting the photosynthetic apparatus and by providing signalling molecules.

## Data Availability

Not applicable.

## Supplementary Materials

The following are available online at https://www.mdpi.com/article/10.3390/ijms22115473/s1, Figure S1: Phylogenetic tree of the SAD from Vitis vinifera, Arabidopsis thaliana, Glycine max and Oryza sativa; Figure S2: Phylogenetic tree of the membrane-bound Δ3 desaturase from Vitis vinifera, Arabidopsis thaliana, Glycine max and Oryza sativa; Figure S3: Phylogenetic tree of the membrane-bound Δ9 desaturase, Δ12/ω6 desaturase, Δ15/ω3 desaturase, sphingolipids Δ4 and the sphingolipid Δ8 desaturases from Vitis vinifera, Arabidopsis thaliana, Glycine max and Oryza sativa; Figure S4: Melting curves of targeted genes: (a) EF1α; (b) UBQ; (c) VviFAD2-1; (d) VviFAD2-2; (e) VviFAD3-1; (f) VviFAD4; G) VviADS; (h) VviFAD6; (i) VviFAD7; (j) VviFAD8; (k) VviSAD-1; (l) VviSAD-2. Table S1: General features of the Vitis vinifera fatty acid desaturases superfamily. Proposed grapevine fatty acid desaturase nomenclature, gene locus, protein, and nucleotide accessions (from NCBI), chromosome location, exon number, protein length, molecular weight (Mw), isoelectric point (pI), and subcellular prediction are represented; Table S2: Cis-element analysis of Vitis vinifera fatty acid desaturases gene promoters; Table S3: Protein motifs of Vitis vinifera fatty acid desaturases; Table S4: Protein domains of Vitis vinifera fatty acid desaturases; Table S5: Subcellular prediction of the Vitis vinifera fatty acid desaturases proteins; Table S6: Reference and target genes transcripts primer sequences, amplicon length, amplification efficiency, annealing, and melting temperature are represented; Table S7: Biological process and function of the Vitis vinifera fatty acid desaturases proteins.
