# Supplementary material for: Fatty Acid Desaturases: Uncovering Their Involvement in Grapevine Defence against Downy Mildew
Source: Int J Mol Sci. 2021 May 22;22(11):5473. doi: 10.3390/ijms22115473 (PMC8196838; doi:10.3390/ijms22115473)
Supplement: Supplementary file 1 [file ijms-22-05473-s001.zip › Supplementary_Figure_S1-4.pdf]

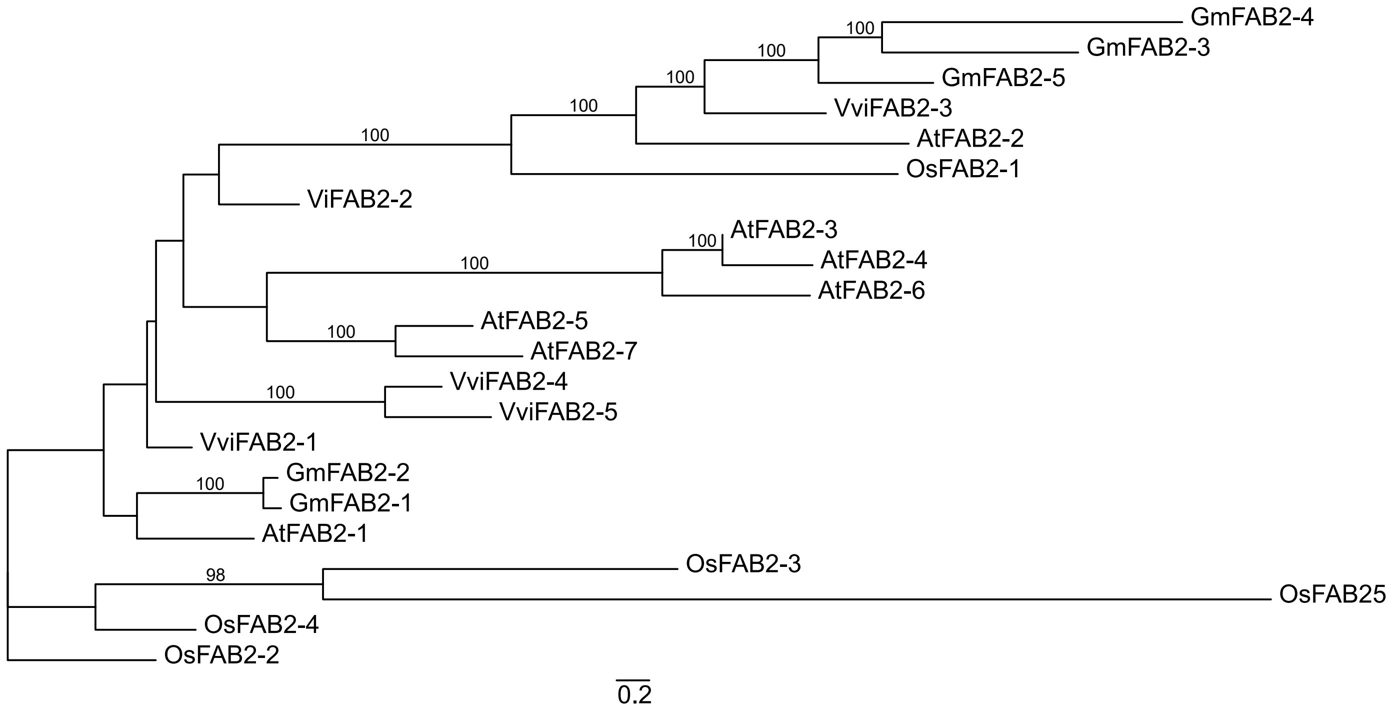

Figure S1 - Phylogenetic tree of the SAD from *Vitis vinifera*, *Arabidopsis thaliana*, *Glycine max* and *Oryza sativa*.

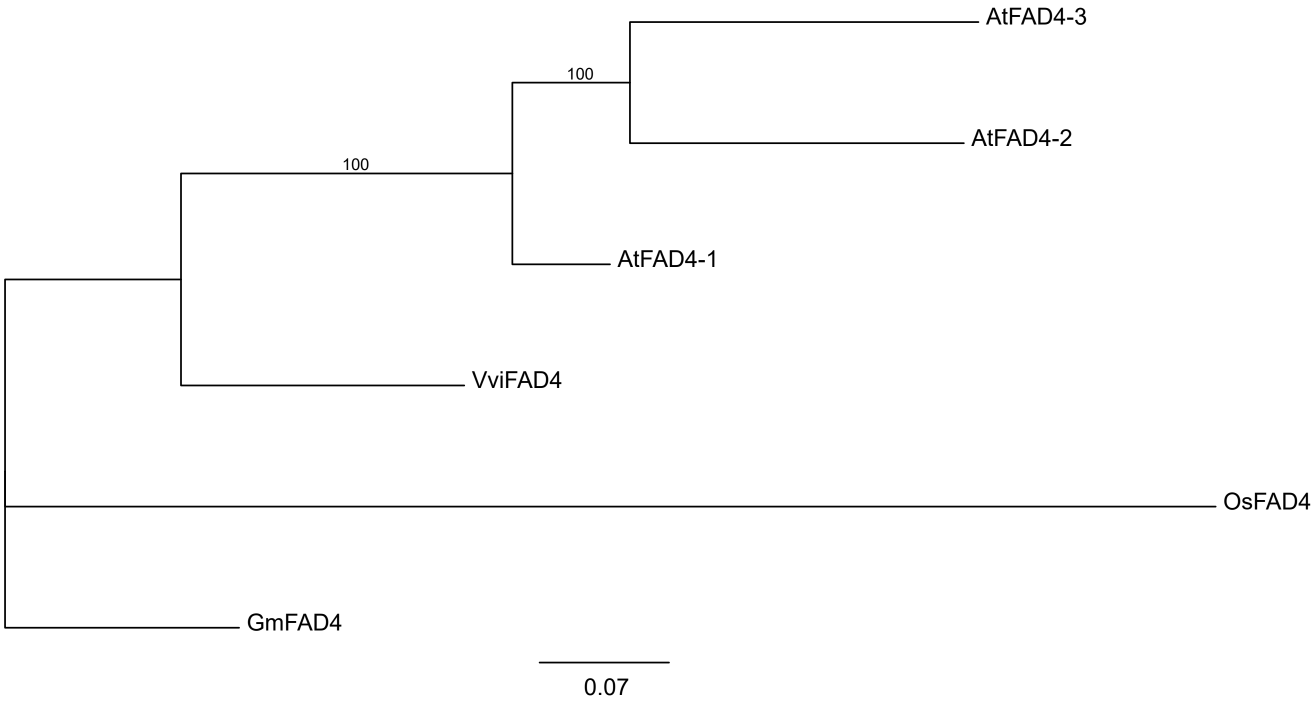

Figure S2 - Phylogenetic tree of the membrane-bound Δ3 desaturase from *Vitis vinifera*, *Arabidopsis thaliana*, *Glycine max* and *Oryza sativa*.

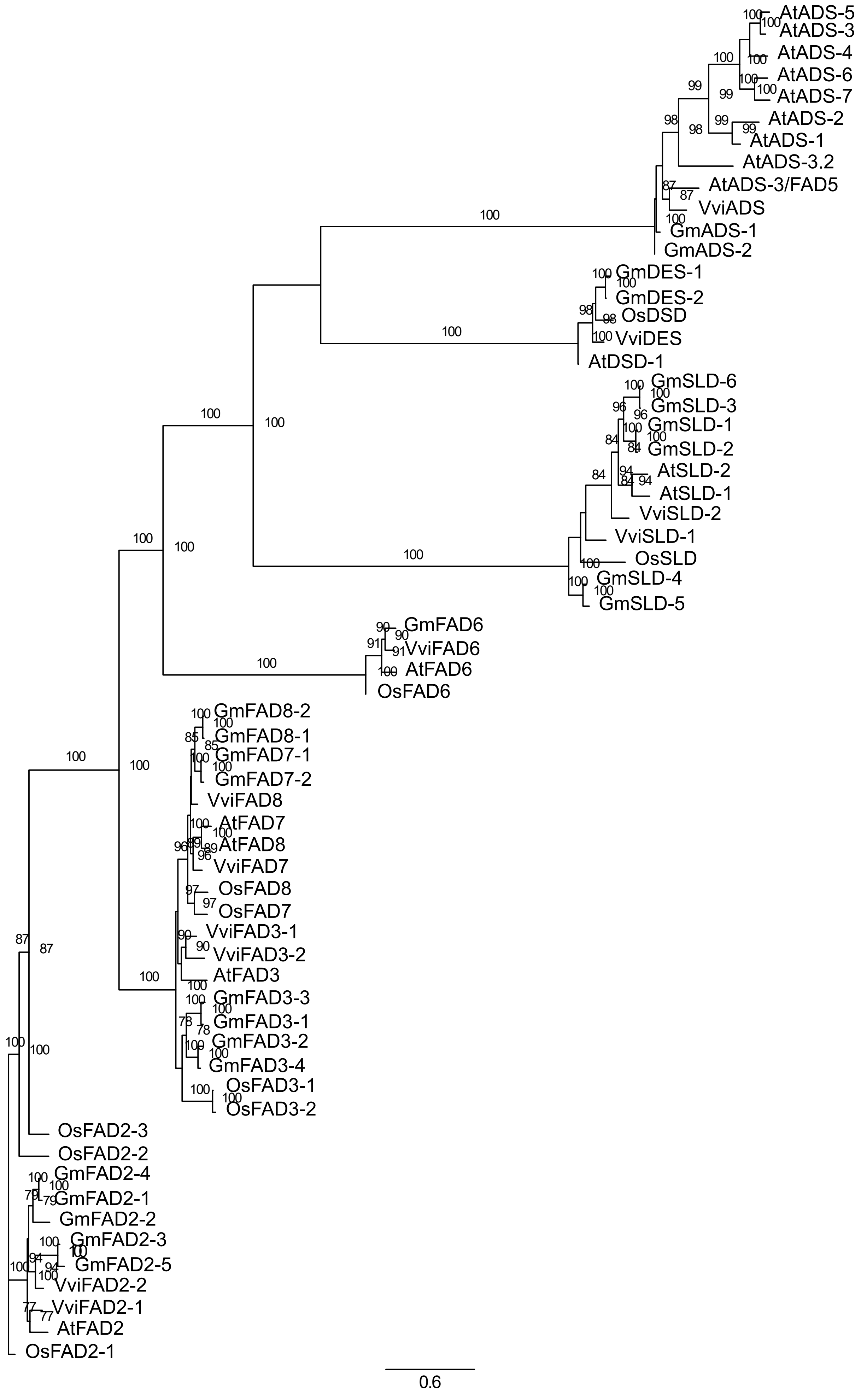

Figure S3 - Phylogenetic tree of the membrane-bound Δ9 desaturase, Δ12/ω6 desaturase, Δ15/ω3 desaturase, sphingolipids Δ4 and the sphingolipid Δ8 desaturases from *Vitis vinifera*, *Arabidopsis thaliana*, *Glycine max* and *Oryza sativa*.

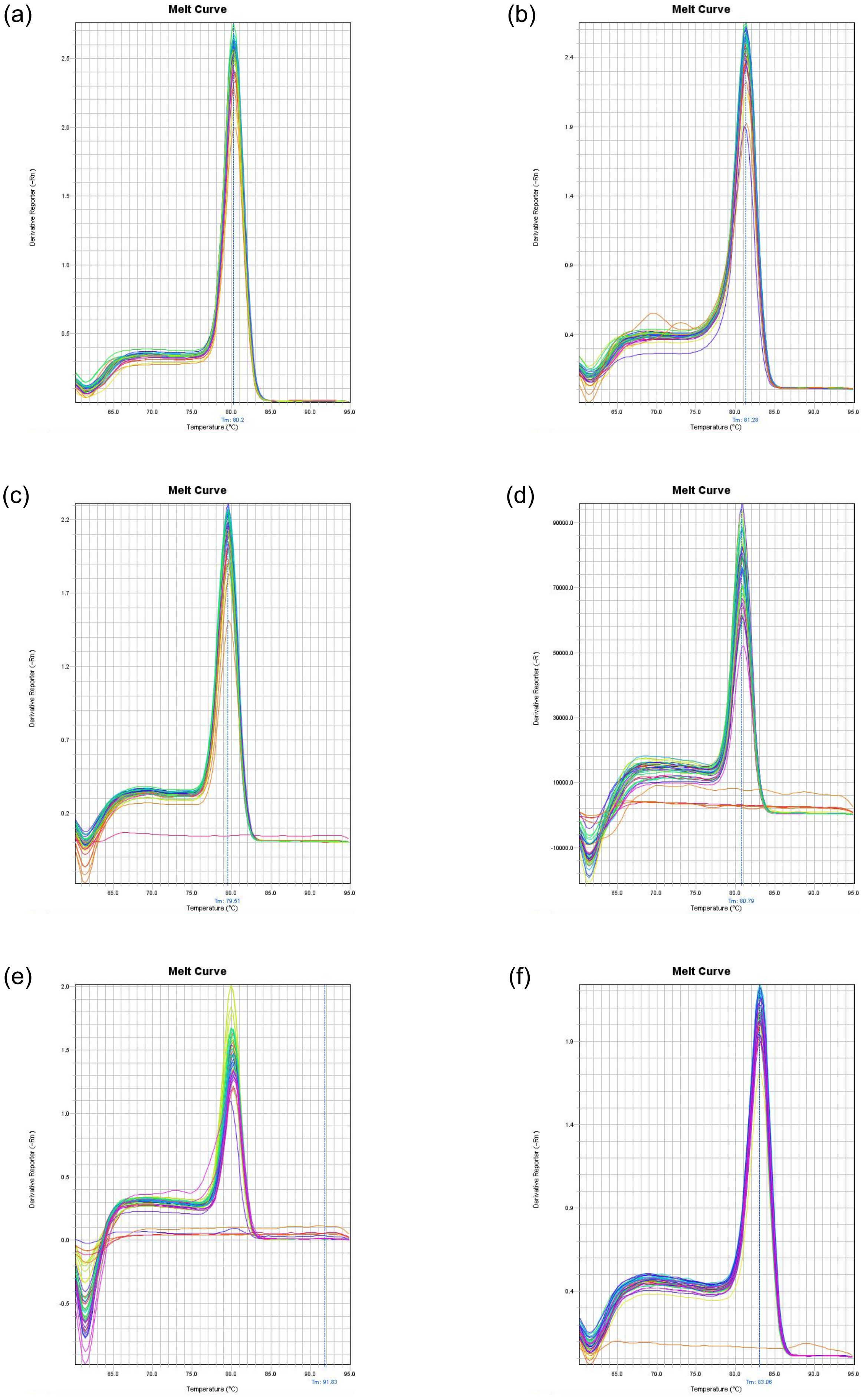

(a)
Melt Curve
Derivative Reporter (-Rn')
Temperature (°C)
Tm: 80.2
(b)
Melt Curve
Derivative Reporter (-Rn')
Temperature (°C)
Tm: 81.28
(c)
Melt Curve
Derivative Reporter (-Rn')
Temperature (°C)
Tm: 79.51
(d)
Melt Curve
Derivative Reporter (-R')
Temperature (°C)
Tm: 80.79
(e)
Melt Curve
Derivative Reporter (-Rn')
Temperature (°C)
Tm: 91.83
(f)
Melt Curve
Derivative Reporter (-Rn')
Temperature (°C)
Tm: 83.06

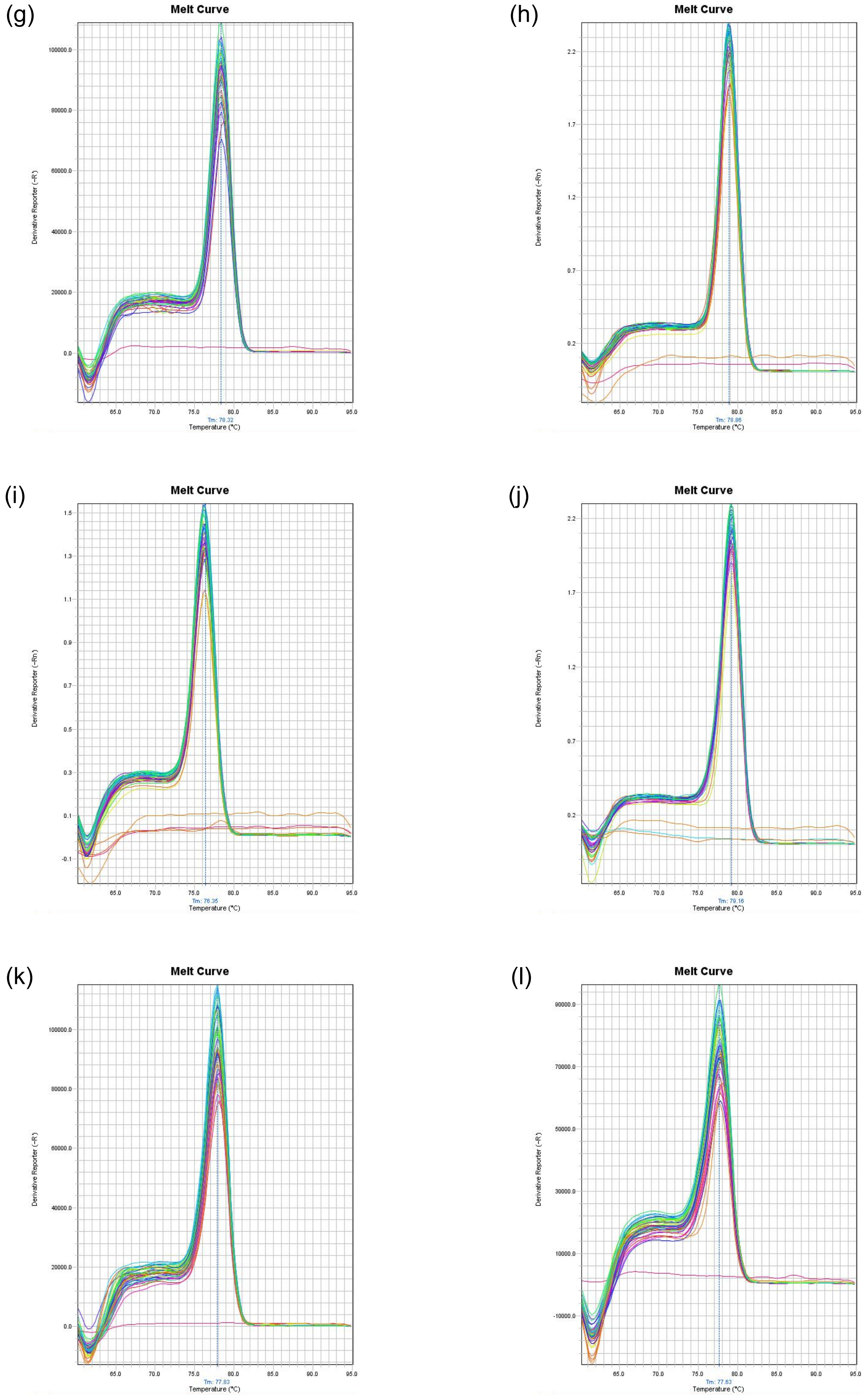

Figure S4 - Melting curves of targeted genes: (a) *EF1α*; (b) *UBQ*; (c) *VviFAD2-1*; (d) *VviFAD2-2*; (e) *VviFAD3-1*; (f) *VviFAD4*; G) *VviADS*; (h) *VviFAD6*; (i) *VviFAD7*; (j) *VviFAD8*; (k) *VviSAD-1*; (l) *VviSAD-2* .
