# Supplementary material for: Fatty Acid Desaturases: Uncovering Their Involvement in Grapevine Defence against Downy Mildew
Source: Int J Mol Sci. 2021 May 22;22(11):5473. doi: 10.3390/ijms22115473 (PMC8196838; doi:10.3390/ijms22115473)
Supplement: Supplementary file 1 [file ijms-22-05473-s001.zip › Supplementary_table_S1_S3-7.pdf]

Supplementary Table S1. General features of *Vitis vinifera* fatty acid desaturases superfamily. Proposed grapevine fatty acid desaturases nomenclature, gene locus, protein and nucleotide accessions (from NCBI), chromosome location, exon number, protein length, molecular weight (Mw), isoelectric point (pI) and subcellular prediction are represented.

Group	Sub-group	Proposed nomenclature	Locus	Nucleotide	Protein	Chr. location	Position on chr. (Mb)	Exons	Protein length (aa)	Mw (kDa)	pI	Subcellular location
Membrane-bound fatty acid desaturases	acyl-lipid Δ12/ω6 desaturase	VviFAD2-1	LOC100252129	XM_002279203.4	XP_002279239.1	8	20.2	2	382	44.05	8.94	Endoplasmic reticulum
		VviFAD2-2	LOC100258789	XM_002285604.3	XP_002285640.1	6	1.4	2	376	43.52	8.83	Endoplasmic reticulum
	acyl-lipid Δ15/ω3 desaturase	VviFAD3-1	LOC100243349	XM_002277537.4	XP_002277573.1	6	7.4	9	386	44.54	8.96	Endoplasmic reticulum
		VviFAD3-2	LOC100251798	XM_010652591.2	XP_010650893.1	6	7.4	9	398	45.97	6.89	Endoplasmic reticulum
	acyl-lipid Δ3 desaturase	VviFAD4	LOC100257229	XM_002280947.4	XP_002280983.1	19	6.3	1	307	34.52	8.52	Chloroplast
	acyl-lipid Δ12/ω6 desaturase	VviFAD6	LOC100248377	XM_003634815.2	XP_003634863.1	11	0.1	10	441	51.13	9.05	Chloroplast
	acyl-lipid Δ15/ω3 desaturase	VviFAD7	LOC100260414	XM_002273738.3	XP_002273774.1	13	0.6	8	456	51.55	8.67	Chloroplast
		VviFAD8	LOC100247097	XM_002264314.4	XP_002264350.1	8	7.7	8	452	51.97	8.57	Chloroplast
	sphingolipids Δ8 desaturase	VviSLD-1	LOC100259521	XM_002279153.4	XP_002279189.1	4	0.99	1	447	51.46	8.56	Endoplasmic reticulum
		VviSLD-2	LOC100265385	XM_002279191.3	XP_002279227.1	15	18.6	1	447	51.03	8.16	Endoplasmic reticulum
	acyl-lipid Δ9 desaturase	VviADS	LOC100249077	XM_002265374.4	XP_002265410.1	9	6.9	5	382	43.77	9.51	Chloroplast
	sphingolipids Δ4 desaturase	VviDES	LOC100854247	XM_003635372.3	XP_003635420.1	un	un	2	327	37.92	6.39	Endoplasmic reticulum
Soluble desaturases	acyl-ACP Δ9 desaturase	VviSAD-1	LOC100242444	XM_002274672.4	XP_002274708.2	5	24.1	3	396	45.37	6.14	Chloroplast
		VviSAD-2	LOC100853715	XM_003635330.3	XP_003635378.1	un	un	3	393	44.99	7.21	Chloroplast
		VviSAD-3	LOC100255873	XM_002264759.4	XP_002264795.1	18	13.6	3	389	44.24	6.16	Chloroplast
		VviSAD-4	LOC100252677	XM_002274616.3	XP_002274652.1	5	24.1	3	387	44.63	6.48	Chloroplast
		VviSAD-5	LOC100267952	XM_002274047.3	XP_002274083.1	5	23.9	3	387	44.30	6.48	Chloroplast

Supplementary Table S3. Protein motifs of the *Vitis vinifera* fatty acid desaturases.

Group	Sub-group	Proposed nomenclature	Histidine-box 1	Start	End	Histidine-box 2	Start	End	Histidine-box 3	Start	End	ER retrieval	Start	End	Cytochrome b5	Start	End	References
Membrane-bound fatty acid desaturases	acyl-lipid Δ12/ω6 desaturase	VviFAD2-1	HECGH	105	109	HRRHH	141	145	HHITDTHVAHH	309	319	YQNKF	378	382				Okuley et al., 1994; McCartney et al., 2004
		VviFAD2-2	HECGH	98	102	HRRHH	134	138	HNITDTHVAHH	302	312	YRNKF	372	376				
	acyl-lipid Δ15/ω3 desaturase	VviFAD3-1	HDCGH	103	107	HRTHH	139	143	HDIGTHVIHH	301	310							Yadav et al., 1993
		VviFAD3-2	HDCGH	115	119	HRTHH	151	155	HNTGTHVIHH	313	322							
	acyl-lipid Δ3 desaturase	VviFAD4	QGHH	152	155	HAWAH	211	215	HAAHH	240	244							Gao et al., 2009
	acyl-lipid Δ12/ω6 desaturase	VviFAD6	HDCAH	165	169	HDRHH	201	205	HYINVHIPHH	356	365							Falcone et al., 1994
	acyl-lipid Δ15/ω3 desaturase	VviFAD7	HDCGH	175	179	HRTHH	211	215	HDIGTHVIHH	373	382							Gibson et al., 1993
		VviFAD8	HDCGH	171	175	HRTHH	207	211	HDIGTHVIHH	369	378							
	sphingolipids Δ8 desaturase	VviSLD-1	HDSGH	158	162	HNAHH	195	199	HGGLQFQIEHH	366	376				HPGG	40	43	Sperling et al., 1998; Li et al., 2012
		VviSLD-2	HDSGH	158	162	HNAHH	195	199	HGGLQFQLEHH	366	376				HPGG	40	43	
	acyl-lipid Δ9 desaturase	VviADS	HRNLSH	155	160	HRYHH	192	196	AFGEGWHNNHH	318	328							Fukuchi-Mizutani et al., 1998
	sphingolipids Δ4 desaturase	VviDES	HELSH	94	98	HLEHH	131	135	WSVGYHNEHH	257	266							Ternes et al., 2002
Soluble desaturases	acyl-ACP Δ9 desaturase	VviSAD-1	ENRH	176	179	EKRH	262	265										Shanklin and Cahoon, 1998
		VviSAD-2	ENRH	173	176	EKRH	259	262										
		VviSAD-3	ENRH	167	170	ERRH	253	256										
		VviSAD-4	ENRH	167	170	EKRH	253	256										
		VviSAD-5	ENRH	167	170	EKRH	253	256										

Supplementary Table S4. Protein domains of the *Vitis vinifera* fatty acid desaturases.

Group	Sub-group	Proposed nomenclature	Domain	Start	End
Membrane-bound fatty acid desaturases	acyl-lipid Δ12/ω6 desaturase	VviFAD2-1	Fatty acid desaturase (PF00487)	82	350
		VviFAD2-2	Fatty acid desaturase (PF00487)	71	343
	acyl-lipid Δ15/ω3 desaturase	VviFAD3-1	DUF3474 (PF11960)	4	73
			Fatty acid desaturase (PF00487)	79	339
		VviFAD3-2	DUF3474 (PF11960)	1	85
			Fatty acid desaturase (PF00487)	91	351
	acyl-lipid Δ3 desaturase	VviFAD4	TMEM189_B_dmain (PF10520)	119	287
	acyl-lipid Δ12/ω6 desaturase	VviFAD6	Fatty acid desaturase (PF00487)	141	397
	acyl-lipid Δ15/ω3 desaturase	VviFAD7	DUF3474 (PF11960)	1	145
			Fatty acid desaturase (PF00487)	150	411
		VviFAD8	DUF3474 (PF11960)	1	141
			Fatty acid desaturase (PF00487)	146	407
	sphingolipids Δ4 desaturase	VviSLD1	Cytochrome b5 (PF00173)	9	101
			Fatty acid desaturase (PF00487)	135	408
		VviSLD2	Cytochrome b5 (PF00173)	9	80
			Fatty acid desaturase (PF00487)	134	408
	acyl-lipid Δ9 desaturase	VviADS	Fatty acid desaturase (PF00487)	130	362
	sphingolipids Δ4 desaturase	VviDES	Sphingolipid Δ-desaturase (PF08557)	11	47
			Fatty acid desaturase (PF00487)	69	297
Soluble desaturases	acyl-ACP Δ9 desaturase	VviSAD-1	Fatty acid desaturase 2 (PF03405)	68	390
		VviSAD-2	Fatty acid desaturase 2 (PF03405)	65	387
		VviSAD-3	Fatty acid desaturase 2 (PF03405)	57	383
		VviSAD-4	Fatty acid desaturase 2 (PF03405)	59	381
		VviSAD-5	Fatty acid desaturase 2 (PF03405)	59	381

Supplementary Table S5. Subcellular prediction of the *Vitis vinifera* fatty acid desaturases proteins.

			TargetP 2.0					Lozalizer Version 1.0.4			Predotar Version 1.04					
**Group**	**Sub-group**	**Proposed nomenclature**	**Other**	**Signal peptide**	**Mitochondiral trasnfer peptide**	**Chloroplast transfer peptide**	**Thylakoid luminal transfer peptide**	**Chloroplast**	**Mitochondria**	**Nucleus**	**Mitochondrial**	**Plastid**	**Endoplasmic Reticulum**	**Elsewhere**	**Result**	**Final Prediction**
**Membrane-bound fatty acid desaturases**	acyl-lipid Δ12/ω6 desaturase	VviFAD2-1	0.9996	0.0001	0	0.0001	0.0002	-	-	-	0,03	0,02	0	0,95	none	Endoplasmic reticulum
		VviFAD2-2	0.9846	0	0.0003	0.0109	0.0041	Y (0.838 | 1-42)	-	-	0,08	0,52	0	0,44	plastid	Endoplasmic reticulum
	acyl-lipid Δ15/ω3 desaturase	VviFAD3-1	1	0	0	0	0	-	-	-	0,01	0,01	0	0,99	none	Endoplasmic reticulum
		VviFAD3-2	1	0	0	0	0	-	-	-	0,01	0,01	0	0,99	none	Endoplasmic reticulum
	acyl-lipid Δ3 desaturase	VviFAD4	0.149	0	0.0179	0.7856	0.0474	-	-	-	0,28	0,26	0	0,53	plastid	Chloroplast
	acyl-lipid Δ12/ω6 desaturase	VviFAD6	0.0685	0.0002	0.0137	0.9009	0.0167	Y (0.971 | 1-25)	-	Y (KRVK)	0,04	0,57	0,02	0,41	plastid	Chloroplast
	acyl-lipid Δ15/ω3 desaturase	VviFAD7	0.0527	0.0001	0.0035	0.9316	0.0122	Y (0.994 | 1-59)	-	-	0,09	0,79	0,01	0,19	plastid	Chloroplast
		VviFAD8	0.201	0	0.0837	0.5356	0.1797	Y (0.996 | 1-40)	-	-	0,02	0,86	0	0,14	plastid	Chloroplast
	sphingolipids Δ8 desaturase	VviSLD-1	0.9999	0	0	0	0	-	-	-	0,01	0	0	0,99	none	Endoplasmic reticulum
		VviSLD-2	1	0	0	0	0	-	-	-	0,01	0	0	0,99	none	Endoplasmic reticulum
	acyl-lipid Δ9 desaturase	VviADS	0.0709	0	0.0005	0.9137	0.0148	Y (1.0 | 1-41)	-	Y (RRRK,RRRR)	0,05	0,93	0,02	0,07	plastid	Chloroplast
	sphingolipids Δ4 desaturase	VviDES	1	0	0	0	0	-	-	-	0,01	0	0	0,99	none	Endoplasmic reticulum
**Soluble desaturases**	acyl-ACP Δ9 desaturase	VviSAD-1	0.013	0	0.0096	0.9761	0.0013	Y (0.828 | 1-42)	Y (0.792 | 1-40)	Y (RKAQDYVCGLAPRFRKL)	0,03	0,33	0,01	0,64	possibly plastid	Chloroplast
		VviSAD-2	0.0724	0	0.0339	0.8759	0.0178	Y (0.999 | 1-26)	-	Y (RKAQDYVCGLAPRIRKL)	0,28	0,88	0	0,09	plastid	Chloroplast
		VviSAD-3	0.0565	0	0.0077	0.932	0.0037	Y (1.0 | 1-41)	-	Y (RRAQDFVCGLAPRIRKL)	0,18	0,05	0,01	0,77	none	Chloroplast
		VviSAD-4	0.0611	0.0104	0.0093	0.9077	0.0114	Y (0.998 | 1-51)	-	Y (RKAQDYVCGLAKRLRT)	0,09	0,63	0,16	0,28	plastid	Chloroplast
		VviSAD-5	0.0489	0.0574	0.0223	0.8552	0.0161	Y (0.999 | 1-51)	-	Y (RKAQDYVCGLAQRLRKL)	0,07	0,62	0,22	0,28	plastid	Chloroplast

Supplementary Table S6. Reference and target genes transcripts primer sequences, amplicon length, amplification efficiency, annealing and melting temperature are represented.

Gene name	Abbreviation	NCBI Accession Number	Primer Sequence	Amplicon length (bp)	Ta (°C)	Tm (°C)	Amplification efficiency (E)	Reference
Elongation Factor 1-alpha	*EF1α*	XM_002284888.3	Fw: AACCAAAATATCCGGAGTAAAAGA<br>Rev: GAACTGGGTGCTTGATAGGC	150	60	80.20	1,95	Reid et al. (2006)
Ubiquitin-conjugating enzyme	*UBQ*	XM_002273532.2	Fw: GTGGTATTATTGAGCCATCCTT<br>Rev: AACCTCCAATCCAGTCATCTAC	182	60	81.28	1,90	Reid et al. (2006)
Membrane-bound fatty acid desaturases	*VviFAD2-1*	XM_002279203.4	FW: CCCAAAACCAAATCCAAACTC<br>Rev: TACTGTAGCGCCTGCCTGAA	139	60	79.51	1,91	
	VviFAD2-2	XM_002285604.3	Fw: GTGCCAAAGCCCAAATCCAG<br>Rev: ATTATAGGGGCCAGACACATT	138	60	80.79	2,04	
	VviFAD3-1	XM_002277537.4	Fw: ATAGAAGCCCAGGGAAGAAG<br>Rev: CAAAGGATACGCAAACAAGCA	135	60	80.00	1,96	
	VviFAD3-2	XM_010652591.2	Fw: TGGTACCGTGGCAAGGAAT<br>Rev: ATGCGTGCCAGTGTTATGTT	Not detected				
	VviFAD4	XM_002280947.4	Fw: TGTTCAGCCAGCAGTTCCAT<br>Rev: CTCGACACTAGCAGTCCAG	97	60	83.06	1,97	
	VviFADS	XM_002265374.4	Fw: TGCGGTGACTGGTCTATTG<br>Rev: AACCCCACAGTAGGCGAAA	109	58	78.32	2,02	
	VviFAD6	XM_003634815.2	Fw: CATGGTTGGGTTATCACTTCT<br>Rev: CTATCCAACGAGGGTAATCAC	147	60	78.86	1,96	
	VviFAD7	XM_002273738.3	Fw: TACCGCTACTAGAACATTG<br>Rev: TCCTACCCCACAGATAAAG	72	54	76.35	1,89	
	VviFAD8	XM_002264314.4	Fw: GGCACTTTCCCTCCTCCTT<br>Rev: GGGCCTTATGCCACATTCT	151	58	79.16	2,04	
Soluble desaturases	*VviSAD-1*	XM_002274672.4	Fw: AGGTCAGGCATACACTCTC<br>Rev: ACGAAAATGCAACTGGGAAAC	89	58	77.83	1,94	
	VviSAD-2	XM_003635330.3	Fw: TATGTCTGTGGACTGGCTC<br>Rev: AGTTGCTTGCTTCACCCTTC	72	58	77.63	1,96	

Supplementary Table S7. Biological process and function of the *Vitis vinifera* fatty acid desaturases proteins.

Group	Sub-group	Proposed nomenclature	Biological process	Putative Function
Membrane-bound fatty acid desaturases	acyl-lipid Δ12/ω6 desaturase	VviFAD2-1	GO:0006629 - lipid metabolic process	None predicted
		VviFAD2-2	GO:0006629 - lipid metabolic process	None predicted
	acyl-lipid Δ15/ω3 desaturase	VviFAD3-1	GO:0006629 - lipid metabolic process<br>GO:0055114 - oxidation-reduction process	GO:0016717 - oxidoreductase activity
		VviFAD3-2	GO:0006629 - lipid metabolic process<br>GO:0055114 - oxidation-reduction process	GO:0016717 - oxidoreductase activity
	acyl-lipid Δ3 desaturase	VviFAD4	None predicted	None predicted
	acyl-lipid Δ12/ω6 desaturase	VviFAD6	GO:0006629 - lipid metabolic process	None predicted
	acyl-lipid Δ15/ω3 desaturase	VviFAD7	GO:0006629 - lipid metabolic process<br>GO:0055114 - oxidation-reduction process	GO:0016717 - oxidoreductase activity
		VviFAD8	GO:0006629 - lipid metabolic process<br>GO:0055114 - oxidation-reduction process	GO:0016717 - oxidoreductase activity
	sphingolipids Δ8 desaturase	VviSLD-1	GO:0006629 - lipid metabolic process	GO:0016491 - oxidoreductase activity
		VviSLD-2	GO:0006629 - lipid metabolic process	GO:0016491 - oxidoreductase activity
	acyl-lipid Δ9 desaturase	VviADS	GO:0006629 - lipid metabolic process<br>GO:0055114 - oxidation-reduction process	GO:0016717 - oxidoreductase activity
	sphingolipids Δ4 desaturase	VviDES	GO:0006629 - lipid metabolic process<br>GO:0030148 - sphingolipid biosynthetic process	GO:0042284 sphingolipid delta-4 desaturase activity
Soluble desaturases	acyl-ACP Δ9 desaturase	VviSAD-1	GO:0006631 fatty acid metabolic process<br>GO:0006633 fatty acid biosynthetic process<br>GO:0055114 oxidation-reduction process	GO:0016491 oxidoreductase activity<br>GO:0045300 acyl-[acyl-carrier-protein] desaturase activity
		VviSAD-2	GO:0006631 fatty acid metabolic process<br>GO:0006633 fatty acid biosynthetic process<br>GO:0055114 oxidation-reduction process	GO:0016491 oxidoreductase activity<br>GO:0045300 acyl-[acyl-carrier-protein] desaturase activity
		VviSAD-3	GO:0006631 fatty acid metabolic process<br>GO:0006633 fatty acid biosynthetic process<br>GO:0055114 oxidation-reduction process	GO:0016491 oxidoreductase activity<br>GO:0045300 acyl-[acyl-carrier-protein] desaturase activity
		VviSAD-4	GO:0006631 fatty acid metabolic process<br>GO:0006633 fatty acid biosynthetic process<br>GO:0055114 oxidation-reduction process	GO:0016491 oxidoreductase activity<br>GO:0045300 acyl-[acyl-carrier-protein] desaturase activity
		VviSAD-5	GO:0006631 fatty acid metabolic process<br>GO:0006633 fatty acid biosynthetic process<br>GO:0055114 oxidation-reduction process	GO:0016491 oxidoreductase activity<br>GO:0045300 acyl-[acyl-carrier-protein] desaturase activity
